# Comparative Evaluation of the Salivary and Buccal Mucosal Microbiota by 16S rRNA Sequencing for Forensic Investigations

**DOI:** 10.3389/fmicb.2022.777882

**Published:** 2022-03-18

**Authors:** Shuangshuang Wang, Feng Song, Haoyu Gu, Xiaowen Wei, Ke Zhang, Yuxiang Zhou, Haibo Luo

**Affiliations:** Department of Forensic Genetics, West China School of Basic Medical Sciences and Forensic Medicine, Sichuan University, Chengdu, China

**Keywords:** oral bacterial community, 16S rRNA, forensic science, high-throughput sequencing, saliva

## Abstract

The human microbiome has emerged as a new potential biomarker for forensic investigations with the development of high-throughput sequencing and bioinformatic analysis during the last decade. The oral cavity has many different microbial habitats, with each habit colonized by specific and individualized microbiota. As saliva and buccal mucosa are common biological evidence in forensic science, understanding the differences of microbial communities between the two is important for forensic original identification. Moreover, the oral microbiota is individualized, whereas there are few studies on the application of forensic personal identification that need to be supplemented. In this study, *Streptococcus* was the most abundant genus, with an average relative abundance of 49.61% in the buccal mucosa, while in the saliva, *Streptococcus*, *Veillonella*, and *Neisseria* had similar proportions (20%, 15%, 16%) and were the dominant genera. The α and β diversity displayed a significant distinctness between the saliva and buccal mucosal groups. The community assembly mechanism stated that the deterministic process played a more significant effect in shaping the salivary bacterial community assembly than buccal mucosa, which explained the microbial differences. Of the test samples, 93.3% can be correctly classified with the random forest model based on the microbial differences. Targeting the low-abundance bacteria at the species level, 52% of experimental participants could be discriminated by using the observed unique bacterial species. In conclusion, the salivary bacterial community composition differed from that of the buccal mucosa and showed high richness and diversity. With the random forest model, the microbiota of saliva and buccal mucosa can be classified, which can be used in identifying the source of oral biological trace. Furthermore, each individual has a unique bacterial community pattern, and the presence or absence of unique bacteria and differences in the composition of the core oral microbiota are the key points for forensic personal discrimination that supplement the study of oral microbial application to forensic personal discrimination. Whether for original identification or personal discrimination, the oral microbiome has great potential for application.

## Introduction

Identifying the origin of biological evidence left at a crime scene and determining the donors are important tasks in forensic practice, which can provide crucial clues during the investigation and evidence for the trial. An increasing number of biological markers have been applied in the two areas. Conventional human genetic markers include short tandem repeats (STRs), single-nucleotide polymorphisms (SNPs), insertions, or deletions (InDels) (Kowalczyk et al., 2018), and the other human cellular biomarkers included RNA (Manetti et al., 2021), methylation (Watanabe et al., 2021), etc. As criminals’ anti-detection capabilities have increased, the human cell may be present at low or undetectable levels, thereby limiting the usefulness of methods based on the human cell. Therefore, forensic scientists have been exploring biomarkers of non-human origin for forensic application. In recent years, the human microbiome has become a new potential biomarker in forensic investigations because it plays a prominent role in human health. Microbial cells colonize various parts of the body and far outnumber the body’s own cells (Turnbaugh et al., 2007), with 10 times more bacterial cells in and on the body than human cells (Human Microbiome and Project Consortium, 2012). Human microorganisms with their nucleic acids are regularly deposited and transferred in a manner similar to human DNA and can be used to identify criminal suspects (Schmedes et al., 2016). Moreover, bacterial DNA is circular and well protected by peptidoglycan, making bacterial DNA more resistant to degradation than human DNA (Kennedy et al., 2012; Leake et al., 2016). On the one hand, the human microbiome is highly individualized (Human Microbiome and Project Consortium, 2012), which makes it possible to apply the human microbiome for forensic personal identification, especially in the case of degraded DNA and low quantities of human DNA. On the other hand, different body habitats each have their own specialized microbiome (Human Microbiome and Project Consortium, 2012), making the human microbiome forensically applied to identifying the origin of biological trace.

Oral biological specimens are crucial evidence in forensic practice. Saliva and mucosa can be left at the crime scene in stains, hickeys, and bite marks that can be detected by policemen for identification. It is crucial to figure out by what activity the biological trace was caused in forensic practice (Hanssen et al., 2018; Quaak et al., 2018). The forensic conventional analysis based on the human cell does not focus on the differences between saliva and oral mucosa, as they both contain oral epithelial cells. However, different microbial habitats are observed in the oral cavity, and each has its own microenvironment that is colonized by different microbial communities (Mark Welch et al., 2020). The differences in the bacterial community between the saliva and buccal mucosa may work well in identifying the origin of oral biological trace in certain cases of special sexual assault involving kissing and other activities with mouth contact. Therefore, it is necessary to determine the differences in microbial community composition between buccal mucosa and saliva. The human oral microbiota is as highly individualized as the skin microbiota, allowing for the application of the oral microbiota for personal identification, especially in the case of degraded DNA and low quantities of human DNA, such as for hickeys and bite marks, which can provide additional criminalistics information on linking oral biological trace to the possible donor. A lot of studies on the human skin microbiome have shown the potential of forensic personal identification (Schmedes et al., 2018; Woerner et al., 2019), whereas similar studies on the oral microbiome are lacking and need to be supplemented.

High-throughput sequencing, as a PCR-based molecular method, has emerged as a common tool for microbial diversity studies. The 16S rRNA gene has become the main marker for amplification sequencing due to its ubiquity and essentiality for the survival of bacteria (Case et al., 2007). To date, high-throughput sequencing of 16S rRNA genes has been frequently used in forensic phylogenetic analyses of microbiomes, including the skin microbiome (Fierer et al., 2010; Wilkins et al., 2017), soil microbiome (Habtom et al., 2019), vaginal microbiome (Zou et al., 2016; Yao et al., 2021), and salivary microbiome (Stahringer et al., 2012; Leake et al., 2016). Moreover, previous studies have used high-throughput sequencing to analyze the salivary microbiome and bacterial DNA amplified from bite marks and teeth (Kennedy et al., 2012; Leake et al., 2016). The method of amplicon sequencing of 16S rRNA genes can be applied to analyze oral microbiome diversity for forensic investigations.

In this study, we aimed to (1) investigate the bacterial community composition in buccal mucosa and saliva based on 16S rRNA gene amplicon sequencing; (2) determine the differences of microbiota in the buccal mucosa and saliva for identifying the origin of biological trace; and (3) supplement the study of using the oral microbiome to differentiate individuals.

## Materials and Methods

### Sample Collection and DNA Extraction

Human saliva and buccal mucosa samples were collected with the approval of the Ethics Committee at the Department of Forensic Genetics, Sichuan University. Samples were obtained from 50 participants aged 20–50 years old who self-declared no history of antibiotic use for 2 months before the study. To assess the microbial signature in the saliva and buccal mucosa samples, we collected both of them for each individual. A total of 100 samples comprising 50 saliva and 50 buccal mucosa swabs were collected. The procedure for sample collection was as follows: (1) saliva was collected by drooling naturally into 1.5-ml sterile EP tubes and then immediately stored at −20°C until DNA extraction; and (2) the buccal mucosa was swabbed by vigorous wiping using a sterile medical swab for 1–2 min, and DNA extraction was performed immediately after natural drying.

A QIAamp ^®^ DNA Mini Kit (QIAGEN, Germany) was used to obtain DNA from the saliva and buccal mucosa samples according to the manufacturer’s protocol. Total DNA was quantified by a NanoDrop 2000c (Thermo Fisher Scientific, Carlsbad, CA, United States) following the manufacturer’s instructions. The DNA was then stored at −20°C until amplification.

### PCR Amplification and Sequencing

Amplification of the V3–V4 region of the 16S rRNA gene was performed using two primers with barcodes: 341F (CCTACGGGAGGCAGCAG) and 806R (CTACCGGGGT ATCTAATCC). The PCR (25 μl) was as follows: 5 μl reaction buffer (5 × ), 5 μl GC buffer (5 × ), 2 μl dNTP (2.5 mM), 1 μl forward primer (10 μM), 1 μl reverse primer (10 μM), 2 μl template DNA (20 ng/μl), 0.25 μl Q5 DNA Polymerase (New England Biolabs, Ipswich, MA, United States), and 8.75 μl nuclease-free water. The thermal cycling conditions for PCR amplification were as follows: initial incubation step at 98°C for 2 min, followed by 30 cycles of denaturation at 98°C for 15 s, annealing at 55°C for 30 s, extension at 72°C for 30 s, and a final extension step at 72°C for 5 min. The purification of amplicon products was performed with VAHTSTM DNA Clean Beads (Vazyme, China) according to the manufacturer’s recommendations to remove any remaining contaminants and PCR artifacts. The quality and quantity of amplicons were confirmed by 1.2% agarose gel electrophoresis and a Quant-iT PicoGreen dsDNA Assay Kit (Invitrogen, Carlsbad, CA, United States). Purified amplicons were used to construct the library according to standard protocols, and sequencing was performed on an Illumina NovaSeq platform (Illumina, San Diego, CA, United States) at Shanghai Personal Biotechnology Co., Ltd. (Shanghai, China).

### Bioinformatics and Statistical Analysis

In this study, the Illumina NovaSeq platform was used for double-end (paired-end) sequencing of community DNA fragments. The sequencing analysis was performed with the QIIME2 (2019.4) pipeline according to the official tutorials^1^ and R package (v3.2.0). DADA2 was used to deprime, quality-filter, denoise, splice, and remove chimeras from raw sequence data (Callahan et al., 2016). First, QIIME2 cutadapt trim-paired was called to excise the primer fragments of the sequences and discard the unmatched primer sequences; then, DADA2 was called by the QIIME2 DADA2 denoise-paired command for quality control, denoising, splicing, and chimera removal. The above steps were analyzed separately for each library. After denoising all libraries, the amplicon sequence variant (ASV) feature sequences and ASV tables were merged, and singleton ASVs were removed at this step. Sequence length distribution statistics was performed for the length distribution of high-quality sequences contained in the full sample. For the feature sequences of each ASV, the Silva database^2^ was used as the reference sequence database for blasting, and the pretrained naive Bayes classifier was used for species annotation in QIIME2 software with default parameters. The ASV and relative abundance tables were leveled using the rarefaction method, and the leveling depth was set to 95% of the minimum sample sequence size. The α-diversity (microbial diversity within a sample) – Chao1, Shannon, Simpson, etc. – was calculated based on the ASV table using QIIME2, and the Shannon indices were plotted as violin boxplots with *t*-tests. The β diversity (microbial diversity between samples) was assessed using Bray–Curtis distances and visualized via non-metric multidimensional scaling (NMDS). A Bray–Curtis distance equal to 1 indicates that the bacterial community composition of an individual is unique and does not share any bacterial community, while a value equal to 0 means that the composition is the same (Toyomane et al., 2021). The random forest model was performed to classify the samples with an R script. The neutral community model was constructed to explore the community assembly mechanism (Chen et al., 2019). The deterministic strength (DS) was calculated based on the null model that referenced previous studies (Santillan et al., 2020).

## Results

### Summary of Sequencing Results

Saliva and buccal mucosa samples were obtained from unrelated healthy individuals, and the total DNA was subsequently subjected to high-throughput sequencing. A total of 10,311,030 raw reads were obtained by high-throughput sequencing of the V3–V4 region of the 16S rRNA gene from 100 samples, with raw read lengths ranging from 50,201 to 199,533 per sample. The length of clean reads ranged from 404 to 428 bp, and 58,252 bacterial ASV sequences were obtained. The rarefaction curve based on the Shannon index of each sample reached a saturation plateau at a sequencing depth of 4,000 as appropriate, as shown in Supplementary Figure 1. The taxonomic composition of the top 10 oral bacteria ranked in relative abundance at the phylum and genus levels are presented in Supplementary Figure 2. *Firmicutes*, *Proteobacteria*, *Bacteroidetes*, *Fusobacteria*, and *Actinobacteria* were the dominant phyla, with average percentages of 58.93, 20.48,12.20, 2.98, and 2.44%, respectively, while *Streptococcus*, *Veillonella*, *Neisseria*, *Haemophilus*, and *Prevotella* were the relatively high abundance bacterial genera, with average shares of 34.55, 16.59, 9.54, 8.49, and 4.48%, respectively. The detailed bacterial community composition of each sample is shown in Supplementary Figure 3.

### Bacterial Community Composition

**FIGURE 1:**
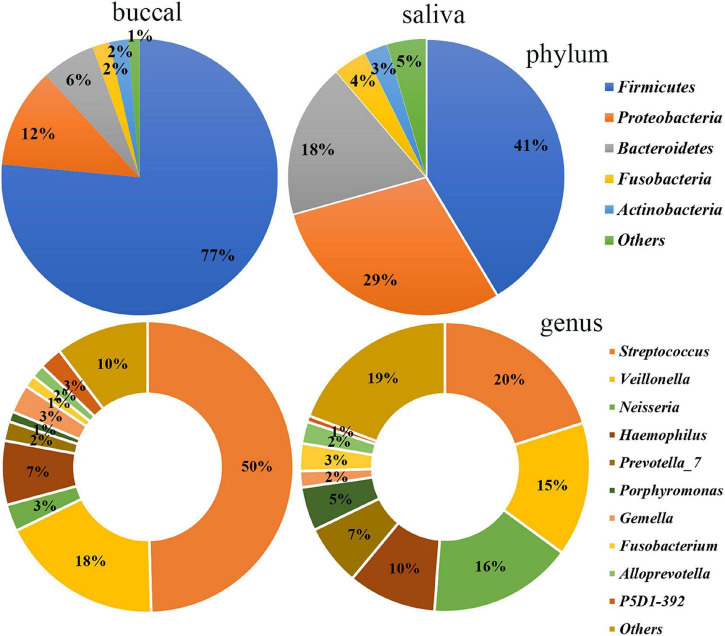
Bacterial community composition of the saliva and buccal mucosa at the phylum and genus levels. At the phylum level, *Firmicutes* was the most abundant in the buccal mucosa and saliva. At the genus level, *Streptococcus* was the most abundant in the buccal mucosa while *Streptococcus*, *Veillonella*, and *Neisseria* had similar proportions in the saliva.

**FIGURE 2:**
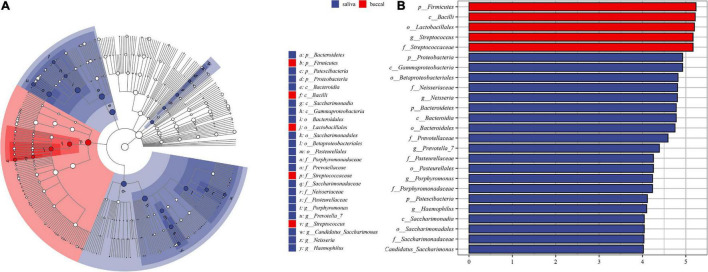
The results of Linear discriminant analysis effect size (LEfSe). **(A)** The cladogram of taxa showed significant differences between saliva and buccal mucosa. **(B)** The bar graph of LDA scores showed the taxa with statistics differences between the two groups. The LDA threshold was 4.

The differences between the saliva and buccal mucosa samples were assessed, with a focus on the composition of bacteria and the relative abundance of each taxon. The numbers of shared and unique ASVs of the saliva and buccal mucosa samples are shown in Supplementary Figure 4. The bacterial community composition at the phylum and genus level of both types of specimens revealed a distinction as shown in Figure 1. In detail, in the buccal mucosa, *Firmicutes* was the most abundant phylum, with an average relative abundance of 76.47%, followed by *Proteobacteria* (11.71%), *Bacteroidetes* (6.29%), *Actinobacteria* (2.19%), and *Fusobacteria* (2.01%), while in saliva, *Firmicutes* (41.39%) was also the most abundant phylum, and other phyla with high relative abundance were *Proteobacteria* (29.36%), *Bacteroidetes* (18.11%), *Fusobacteria* (3.94%), and *Actinobacteria* (2.70%). Moreover, in the buccal mucosa, *Streptococcus* was the most abundant genus, with an average relative abundance of 49.61%, while in the saliva, *Streptococcus*, *Veillonella*, and *Neisseria* had similar proportions (20%, 15%, 16%) and were the dominant genera. Bacteria with significant differences at the phylum and genus levels are displayed in Table 1, and the mean relative abundance and *p* values of the top 10 oral bacteria were calculated. Seven phyla and nine genera showed significant differences (*p* < 0.05) between the saliva and buccal mucosa samples. A species variability analysis was performed, which identified significant differences of 16 species in the saliva and four in the buccal mucosa (Supplementary Figure 5). From the results of a Linear discriminant analysis Effect Size (LEfSe), a total of 25 nested taxa were identified, which explained the differences, with 20 nested taxa in the saliva and five in the buccal mucosa. As shown in Figure 2, the saliva group was characterized by the phyla *Bacteroidetes, Patescibacteria*, and *Proteobacteria*, while the buccal mucosa group was characterized by the phylum *Firmicutes*.

**TABLE 1:** Relative abundance^a^ of taxa differently distributed between saliva and buccal mucosa.

		Saliva	Buccal mucosa	*P*-value
phylum	*Firmicutes*	0.4139 (±0.0240)	0.7647 (±0.0204)	<0.0001, ***
	*Proteobacteria*	0.2926 (±0.0210)	0.1171 (±0.0146)	<0.0001, ***
	*Bacteroidetes*	0.1811 (±0.0169)	0.0629 (±0.0108)	<0.0001, ***
	*Fusobacteria*	0.0394 (±0.0044)	0.0201 (±0.0031)	<0.0001, ***
	*Actinobacteria*	0.0270 (±0.0039)	0.0219 (±0.0036)	0.0652, ns
	*Patescibacteria*	0.0286 (±0.0045)	0.0029 (±0.0006)	<0.0001, ***
	*Spirochaetes*	0.0043 (±0.0012)	0.0011 (±0.0004)	<0.0001, ***
	*Epsilonbacteraeota*	0.0043 (±0.0006)	0.0008 (±0.0002)	<0.0001, ***
	*Synergistetes*	0.0021 (±0.0006)	0.0013 (±0.0005)	0.1167, ns
	*Tenericutes*	0.0007 (±0.0003)	0.0001 (±0.00004)	0.1308, ns
genus	*Streptococcus*	0.2008 (±0.0231)	0.4961 (±0.0315)	<0.0001, ***
	*Veillonella*	0.1499 (±0.0177)	0.1820 (±0.0231)	0.467, ns
	*Neisseria*	0.1609 (±0.0176)	0.0299 (±0.0064)	<0.0001, ***
	*Haemophilus*	0.0987 (±0.0107)	0.0712 (±0.0126)	0.0042, **
	*Prevotella_7*	0.0682 (±0.0120)	0.0214 (±0.0070)	<0.0001, ***
	*Porphyromonas*	0.0488 (±0.0083)	0.0114 (±0.0023)	<0.0001, ***
	*Gemella*	0.0181 (±0.0038)	0.0316 (±0.0050)	0.0017, **
	*Fusobacterium*	0.0308 (±0.0041)	0.0133 (±0.0024)	<0.0001, ***
	*Alloprevotella*	0.0243 (±0.0036)	0.0147 (±0.0044)	0.0004, ***
	*P5D1-392*	0.0069 (±0.0008)	0.0251 (±0.0021)	<0.0001, ***

^a^Relative abundance expressed as the mean value and standard error. Unless otherwise described, ±in this study represents the standard error. **represents P < 0.01 and ***represents P < 0.001.

### Bacterial Community Diversity

**FIGURE 3:**
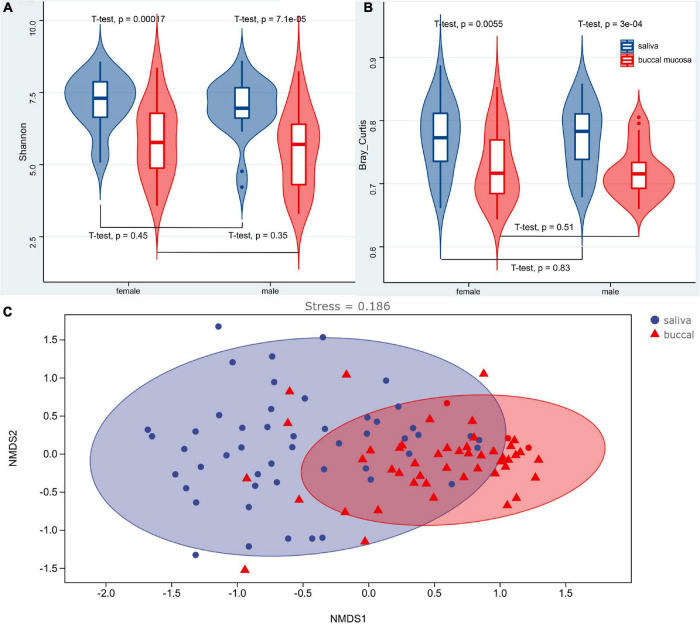
Violin boxplot of the Shannon index **(A)**, Bray–Curtis distance **(B)**, and the NMDS analysis **(C)** of the saliva and buccal mucosa. A significant difference (*p* < 0.05) was observed between the saliva and buccal mucosa groups, while variations were not observed with regard to sex (*p* > 0.05). NMDS analysis based on the Bray–Curtis distance showed dense clustering in the buccal mucosa but a more dispersed pattern in the saliva without strong clustering, and overlaps were observed between the saliva and buccal mucosa groups.

The α and β diversity was calculated, which are shown in Supplementary Tables 1, 2. Richness and diversity were characterized by the Shannon index. The Bray–Curtis distance was assessed to characterize the β diversity. The mean values of the Shannon index for the saliva and buccal mucosa samples were 7.0760 (±0.1328) and 5.6843 (±0.1883), respectively. The violin boxplot of α and β diversity was shown in Figures 3A,B. A significant difference (*p* < 0.05) was observed between the saliva and buccal mucosa groups, while variations were not observed with regard to sex (*p* > 0.05). A Spearman correlation test was performed for the Shannon index and the age of subjects, and it showed no significant correlation between the Shannon index and subject age in the salivary group and buccal mucosal group (saliva: *R* = 0.087, *p* = 0.59; buccal mucosa: *R* = −0.0037, *p* = 0.98) (Supplementary Figure 6A). It also denoted no significant correlation between Bray–Curtis distance and subjects’ age in two experimental groups (saliva: *R* = 0.26, *p* = 0.11; buccal mucosa: *R* = 0.19, *p* = 0.23) (Supplementary Figure 6B). A non-metric multidimensional scaling (NMDS) analysis based on the Bray–Curtis distance was performed to determine the differences among samples, and it showed dense clustering in the buccal mucosa but a more dispersed pattern in the saliva, and overlaps were observed between the saliva and buccal mucosa groups (Figure 3C).

### Bacterial Community Assembly

**FIGURE 4:**
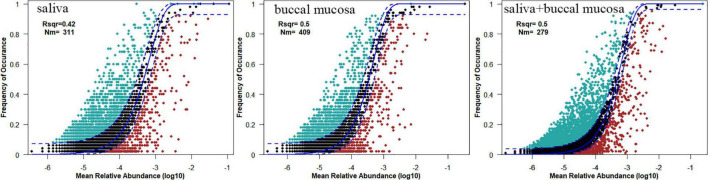
The neutral community model of community assembly. The solid blue lines represented the fittest to the model, while the dashed blue lines represented 95% confidence intervals. Cyan, black, and red plots represented the occurrence frequency of OTUs above prediction, fit prediction, and below prediction, respectively. R^2^ remarked the fitness of the neutral community model.

To evaluate the influence of various oral habitats on bacterial community and subcommunity assembly, the neutral community model was performed to show the relationship between the relative abundance of ASVs and frequency of occurrence. The R square represents the overall goodness of fit of the neutral community model, with 0.42 in saliva, 0.5 in the buccal mucosa, and 0.5 in saliva and mucosa (Figure 4). The Nm value was higher for the bacterial taxon in buccal mucosa than saliva. To further explore the assembly mechanism, the deterministic strength (DS) was calculated based on the null model, which is a metric of deterministic assembly (Santillan et al., 2020). The DS was higher for saliva than buccal mucosa, with both of them below 50%, remarking stronger stochasticity (Table 2).

**TABLE 2:** The diversity and deterministic strength (DS) of saliva and buccal mucosa that output from the null model.

Sample type	Gamma	Obs.mean.alpha	Obs.beta	Mean.null.beta	Ses.beta	DS
Saliva	37377.00	1535.20	0.96	0.74	2131.38	23.14
Buccal mucosa	280048.00	1225.02	0.96	0.81	1421.63	15.32

### Random Forest Classification

**FIGURE 5:**
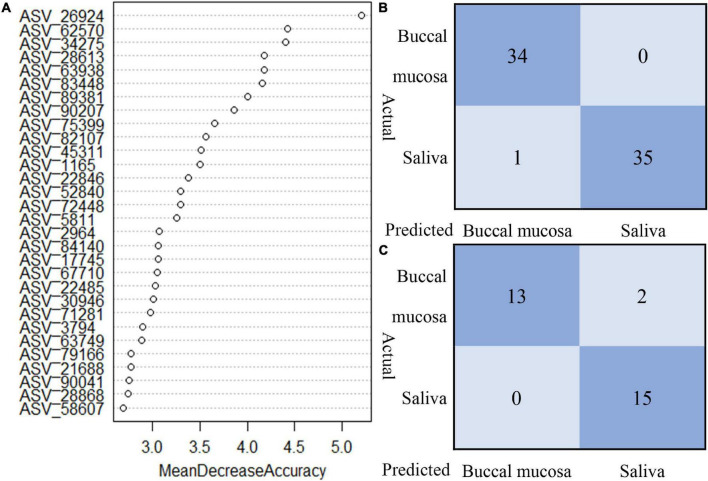
The random forest model to classify the origin of sample type. **(A)** Top 30 important ASVs to perform the prediction. **(B)** Train data showed 98.57% of true predictions represented the good fitness of the model. **(C)** Test data showed 93.33% of true prediction, with mismatching of two samples in test data.

The random forest model was performed to classify the origin of the sample type. Based on the data of relative abundance of ASVs, 70% of the data are divided into a training set and 30% into a test set. The importance score for each ASV was calculated, and we selected the top 30 important ASVs to perform the prediction. As shown in Figure 5, the test of train data showed that 98.57% of true prediction represented the good fitness of the model; 28 samples were correctly classified, with mismatching of two samples in test data.

### Bacterial Community Composition of Individuals

**FIGURE 6:**
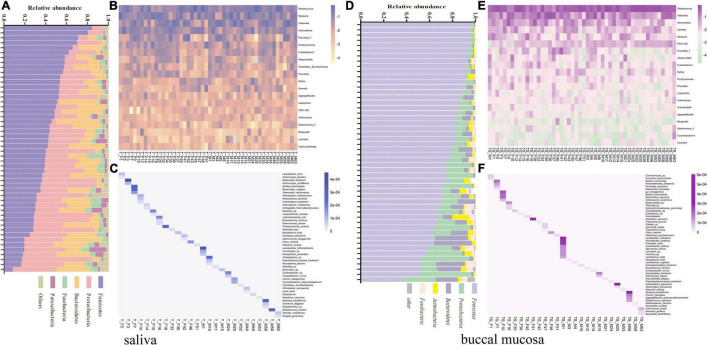
**(A)** Relative abundance of the five major bacterial phyla of all individual saliva samples, sorted by decreasing *Firmicutes* content. **(B)** Heatmap of the top 20 bacterial genera of all individual saliva samples according to the raw relative abundance. **(C)** Heatmap of the relative abundance of 47 specific bacterial species among 26 individual saliva samples according to the raw abundance value. **(D)** Relative abundance of the five major bacterial phyla of all individual buccal mucosal samples, sorted by decreasing *Firmicutes* content. **(E)** Heatmap of the top 20 bacterial genera of all individual buccal mucosal samples according to the raw relative abundance. **(F)** Heatmap of the relative abundance of 47 specific bacterial species among 26 individual buccal mucosal samples according to the raw abundance value.

The saliva and buccal mucosal bacterial community composition were investigated separately among unrelated healthy individuals. To characterize the differences among each subject, the unique bacterial taxa and various relative abundances of the core microbiome were analyzed simultaneously. The relative abundance of the five major bacterial phyla and the bacterial community heatmap for the top 20 bacterial genera are shown in Figures 6A,B,D,E, which presents a specific taxon composition for each individual. At the species level, the bacterial taxa with a low relative abundance ranked outside 100 were targeted to identify unique bacterial species in each individual. A total of 47 and 52 specific bacterial species were observed in the salivary microbiota and buccal mucosal microbiota in 26 subjects, respectively (Figures 6C,F). Fifty-two percent of experimental participants could be discriminated by these observed unique bacterial species. Except for these specific bacterial taxa, the bacterial taxa found in all individuals were investigated, with a focus on the relative abundance of the taxa. A series of 16 bacterial genera that were present in all subjects’ oral cavities (core oral microbiome) were found, and they included *Streptococcus, Veillonella, Neisseria, Haemophilus, Prophyromonas, Gemella, Fusobacterium, Rothia, Prevotella, Aggregatibacter, Leptotrichia, Actinomyces, Granulicatella, Lautropia, Corynebacterium*, and *Capnocytophaga.* The relative proportion was recalculated based on the 16 bacterial genera. The details are shown in Supplementary Table 3, which revealed that a unique taxon composition occurred in each individual.

## Discussion

Conventional forensic analyses based on human cells are not focused on the differences between saliva and oral mucosa, which both contain oral epithelial cells. However, the situation is different when the oral microbiome is applied to forensic identification. Various oral habitats have diverse microbial communities due to the distinct microenvironments (Mark Welch et al., 2020). Therefore, the variations in the salivary and buccal mucosal microbiota of unrelated healthy individuals were investigated to determine their potential forensic applications in this study. Moreover, considering that the DNA profile may be limited in the case of degraded DNA or low human DNA amounts or twins, the potential of applying oral microbiota to forensic personal identification was analyzed to supplement research in this area.

### Differences of Salivary and Buccal Mucosal Microbiota

In the human oral cavity, *Firmicutes*, *Proteobacteria*, *Bacteroidetes*, *Fusobacteria*, and *Actinobacteria* were the dominant phyla that are consistent with previous oral microbiota studies (Stahringer et al., 2012; Moon and Lee, 2016; van der Meulen et al., 2018; Abdulhaq et al., 2021). *Firmicutes* was the most abundant phylum in the saliva, although its mean relative abundance was significantly lower than that in the buccal mucosa (*p* < 0.0001). Another study revealed the same results and showed that the proportion of *Firmicutes* in the buccal mucosa was higher than that in the saliva, both in the disease and control cohort (Kim et al., 2016). The results of the differences in the relative proportion of bacterial taxa were further supported by the LEfSe analyses. *t*-Tests with *p* < 0.0001 indicated a significant difference in the α and β diversity in saliva and buccal mucosa. According to the study by Caselli et al. (2020), the α values of the saliva were higher than those of the buccal mucosa, which was consistent with our study and indicated that the composition of the microbial community in the saliva was richer and more diverse than that in the buccal mucosa. However, the NMDS analysis based on the Bray–Curtis distance displayed some degree of overlap between the saliva and buccal mucosa samples, suggesting that the bacterial community composition of saliva and buccal mucosa were not completely incompatible and that they shared some bacterial communities, as seen from the community composition analysis above. The microbial differences may be attributed to gradients and variations in the physicochemical characteristics of different locations in the oral cavity, and the environmental factors that can influence the distribution of bacteria could be oxygen and pH (Simon-Soro et al., 2013). The community assembly mechanism was assessed to explore the differences. The results of the neutral community model indicated that the community construction of saliva was hardly influenced by the stochastic process and more likely influenced by the deterministic process, while the buccal mucosal community assembly was opposite. That is also proven by the higher DS value in saliva than buccal mucosa. A higher DS value indicates a stronger effect of deterministic assembly of mechanism, while a lower DS value that denotes the effect of stochastic-based assembly mechanism is more significant (Santillan et al., 2020). The overall Nm value was relatively low in our study, compared with another study of the environmental microbiome (Chen et al., 2019), suggesting that species dispersal in the oral community is restricted. The community assembly mechanism stated that the deterministic process played a more significant effect in shaping the salivary bacterial community assembly than buccal mucosa, which may be one of the other explanations for the microbial differences. Combined with our results and those of previous studies (Zaura et al., 2009; Kim et al., 2016; Yu et al., 2017; Stehlikova et al., 2019; Caselli et al., 2020; Zhou et al., 2021; Table 3), we defined several bacterial taxa as core oral bacteria that were present in the saliva and buccal mucosa of all individuals regardless of the 16S rRNA gene variable regions, sequencing techniques, and reference databases. These core oral bacteria served as a crucial role in the identification of the source of body fluids in forensic practice (Hanssen et al., 2018).

**TABLE 3:** Comparison of the saliva and buccal mucosa microbiota composition of healthy individuals.

References	Variable region	Reference database	Saliva	Buccal mucosa
Zhou et al., 2021	V4–V5	SILVA	*Streptococcus, Neisseria, Prevotella, Porphyromonas, Haemophilus, Veillonella, Fusobacterium, Alloprevotella, Pseudomonas, Rothia, Gemella*	
Caselli et al., 2020	whole genome sequencing		*Streptococcus, Neisseria, Prevotella, Haemophilus, Rothia, Actinomyces, Veillonella, Fusobacterium, Corynebacterium, Capnocytophaga, Aggregatibacter, Gemella, Lautropia, Simonsiella, Schaalia, Mogibacterium, Pseudopropionibacterium, Tannerlla, Campylobacter, Cardiobacterium, Leptotrichia*	
Stehlikova et al., 2019	V3–V4	eHOMD		*Streptococcus, Haemophilus, Gemella, Neisseria, Prevotella, Bergeyella, Veillonella, Porphyromonas, Granulicatella, Rothia, Fusobacterium*
Yu et al., 2017	V3–V4	Greengenes	*Streptococcus, Veillonella, Prevotella, Haemophilus, Actinomyces, Rothia, Leptotrichia, Gemella, Lactobacillus*	
Kim et al., 2016	V1–V3	EzTaxon	*Streptococcus, Haemophilus, Neisseria, Rothia, Veillonella, Prevotella, Fusobacterium, Lautropia, Campylobacter, Actinomyces, Capnocytophaga, Leptotrichia, Gemella, Porphyromonas*	*Streptococcus, Haemophilus, Rothia, Neisseria, Actinomyces, Veillonella, Lautropia, Fusobacterium, Escherichia, Gemella, Prevotella, Corynebacterium, Granulicatella, Leptotrichia, Capnocytophaga*
Zaura et al., 2009	V5–V6	SILVA	*Streptococcus, Neisseria, Corynebacterium, Rothia, Actinomyces, Haemophilus, Prevotella, Fusobacterium, Granulicatella, Capnocytophaga*	
This study	V4–V3	SILVA	*Streptococcus, Veillonella, Neisseria, Haemophilus, Porphyromonas, Gemella, Fusobacterium, Alloprevotella, Rothia, Prevotella, Candidatus_Saccharimonas, Aggregatibacter* *Leptotrichia, Actinomyces, Selenomonas_3, Granulicatella, Bergeyella, Lautropia*	

### Potential of the Oral Microbial Differences for the Forensic Original Identification

In recent forensic investigations, the sole purpose is no longer to find the donor of the trace material; rather, identifying the activity that generates biological trace has become an important task (Hanssen et al., 2018; Quaak et al., 2018). Core bacteria and cell types with oral specificity can be useful to identify the origin of body fluids. Nevertheless, the identification of oral epithelial cells or core oral bacteria cannot yet fully reproduce a crime scene or identify whether activities such as kissing or oral molestation have occurred in some specific sexual cases. The differences of bacterial community between saliva and buccal mucosa maybe play essential roles in these cases. From a microbiological point of view, kissing and other mouth contact activities deposit trace material, which mostly consists of oral mucosal microbiota or a mixture of salivary and mucosal microbial communities; therefore, evaluating the mucosal or (and) salivary microbiota is the key for reconstructing crime scene activity. With the random forest classification model based on the microbial differences, 93.3% of samples can be correctly classified into saliva and buccal mucosal groups, which indicated that the microbiota worked well in the identification of biological traces from different oral origins, while the other detection methods based on human DNA cannot achieve such performance.

### Potential of the Oral Microbiota for Forensic Personal Discrimination

We hypothesized that several bacteria are useful for personal discrimination using the oral microbiota because they may be strongly associated with an individual and unique to that individual. Focusing on the bacterial species level, we found several specific bacterial species in the saliva and buccal mucosa of 26 subjects. Fifty-two percent of experimental participants could be discriminated with these unique bacterial species. From our results, the series of specific bacterial species all presented low abundance. Thus, low-abundance bacterial taxa may be more useful for discriminating individuals than high-abundance taxa. However, due to the specificity of bacterial taxa in low abundance, they may be susceptible to lose from the original microbiota and deposited traces, and they are also not easily deposited on a surface as biological traces (Franzosa et al., 2015; Wilkins et al., 2017). Thus, the bacterial taxa that were present in both saliva and mucosa (defined as “core oral bacteria”) in high abundance were analyzed. The relative proportion of core oral bacterial members in each individual constituted a particular taxon composition that has the potential to discriminate subjects. The percentage of the 16 core oral bacterial genera is the microbial code of each individual, similar to an STR profile. This composition varies among individuals and may be able to distinguish various subjects by the different microbial codes. Moreover, it is possible to perform microbial matching with a “reference microbiota code database”; nevertheless, additional research on oral microbiota is required to construct such a database. The main discrepancies in the core microbiome were the relative abundance. However, the factors affecting the abundance of bacterial taxa are numerous. The influence of environmental factors and differences in the processing of multiple steps including extraction, amplification, sequencing, and bioinformatics analysis (Khachatryan et al., 2020) can lead to discrepancies in the abundance of bacterial taxa in the final results. Environmental factors usually are controlled in research as much as possible, but it is highly unpredictable in actual forensic practice; additionally, the differences caused by experimental treatments do not facilitate the comparison of results among laboratories. These are the main challenges in applying microbial taxa for personal identification, which need to be addressed by more research. A series of other techniques included amplificon sequencing of the Clustered Regularly Interspaced Short Palindromic Repeats (CRISPR) gene and intergenic spacer region (ISR); metagenomic and meta-transcriptomic sequencing also shows a potential use in forensic identification except 16S rRNA gene sequencing. Some researchers had explored the possibility of applying the CRISPR gene (Toyomane et al., 2021) and the ISR (Mukherjee et al., 2018) to personal identification. As to metagenomic and meta-transcriptomic sequencing, various studies are focusing on human disease. The metagenomes and meta-transcriptomes of oral microbiota showed differences between disease with periodontitis and dental caries and health groups (Belstrom et al., 2017), while each subject within the two cohorts had a unique microbial composition. As entire genes are sequenced, a higher resolution may be revealed. Furthermore, previous studies have demonstrated that oral disease is associated with species-specific gene expression of the oral microbiota (Belstrom et al., 2021), suggesting that targeting oral-specific active species may play a vital role in personal identification, like the forensic application of *Propionibacterium acnes* in the skin microbiota (Yang et al., 2019). Additional studies should focus on constructing a standardized detection method and statistical analysis to promote forensic identification based on the human oral microbiome.

## Conclusion

In summary, we explored bacterial community compositions in the saliva and buccal mucosa for application to forensic investigations. The results of 16S rRNA sequencing indicated that the bacterial community composition in saliva differed from that of the buccal mucosa at the phylum, genus, and species levels, and higher abundance and diversity were observed in the saliva. The differences in the bacterial community between the saliva and buccal mucosa play an important role in the reconstruction of crime scenes for certain special sexual assault cases involving kissing and other activities with mouth contact. With the random forest model based on microbial differences, the microbiota of saliva and buccal mucosa can be classified. Furthermore, each individual has a unique oral bacterial community pattern, and the presence or absence of unique bacteria and differences in the composition of the core oral microbiota are the key points to discriminate individuals, which supplement the study of oral microbial application to forensic personal discrimination. Whether for original identification or personal discrimination, the human oral microbiome has great potential for forensic investigations.

## Data Availability Statement

The original contributions presented in the study are publicly available. This data can be found here: PRJNA765405.

## Ethics Statement

The studies involving human participants were reviewed and approved by the Ethics Committee at the Department of Forensic Genetics, Sichuan University. The patients/participants provided their written informed consent to participate in this study.

## Author Contributions

HL conceptualized the work. SW, FS, and HL designed the study. HG and YZ collected the samples. HG, XW, and KZ extracted the genomic DNA. SW performed the sequencing and the bioinformatic data analysis. SW and FS wrote and edited the manuscript. All authors read and approved the final manuscript.

## Conflict of Interest

The authors declare that the research was conducted in the absence of any commercial or financial relationships that could be construed as a potential conflict of interest.

## Publisher’s Note

All claims expressed in this article are solely those of the authors and do not necessarily represent those of their affiliated organizations, or those of the publisher, the editors and the reviewers. Any product that may be evaluated in this article, or claim that may be made by its manufacturer, is not guaranteed or endorsed by the publisher.

## Footnotes

1
https://docs.qiime2.org/2019.4/tutorials/

2
https://www.arb-silva.de/
