# Supplementary material for: Comparative Evaluation of the Salivary and Buccal Mucosal Microbiota by 16S rRNA Sequencing for Forensic Investigations
Source: Front Microbiol. 2022 Mar 18;13:777882. doi: 10.3389/fmicb.2022.777882 (PMC8971900; doi:10.3389/fmicb.2022.777882)
Supplement: Supplementary file 1 [file Data_Sheet_1.docx]

Supplementary Material

#
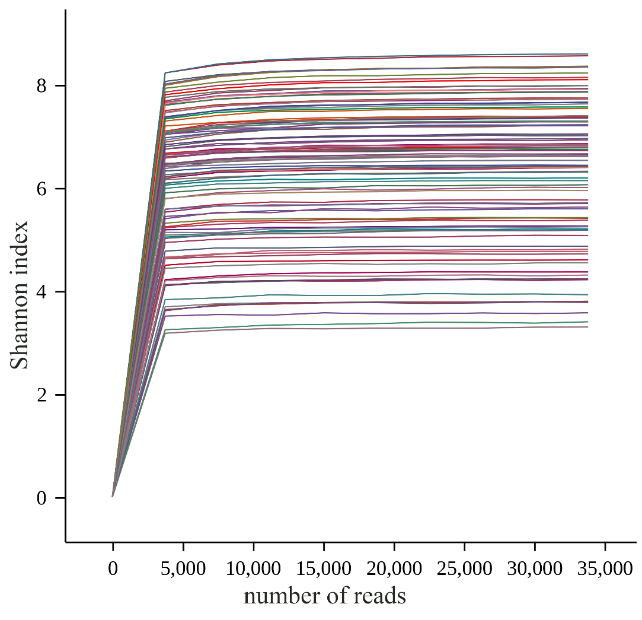
Supplementary Figures


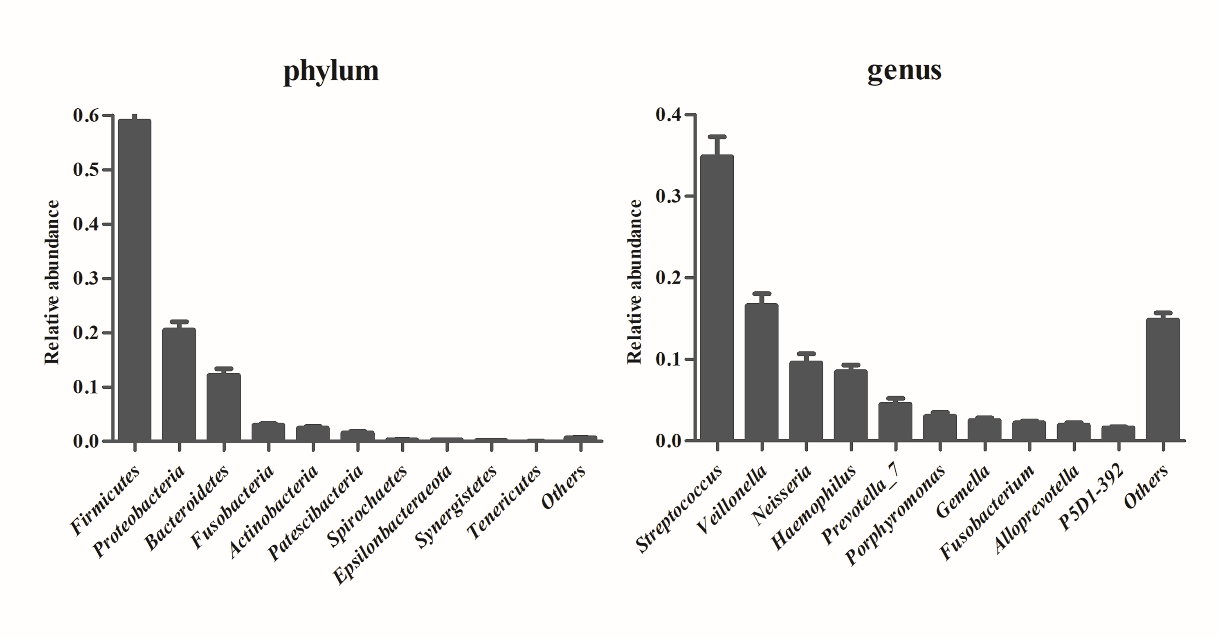
 **Supplementary Figures 1**  Rarefaction curve based on the Shannon index of each sample reached a saturation plateau at a sequencing depth of 4000, as appropriate. The Shannon index can represent the species richness within a sample, and reaching saturation means that increasing the sequencing depth did not lead to increases in the observed species.

**Supplementary Figures 2** Top 10 bacterial taxa in terms of relative abundance at the phylum and genus levels. At the phylum level, *Firmicutes*, *Proteobacteria*, *Bacteroidetes*, *Fusobacteria*, and *Actinobacteria* were the dominant bacterial taxa. At the genus level, *Streptococcus*, *Veillonella*, *Neisseria*, *Haemophilus*, and *Prevotella* showed the highest relative abundance.


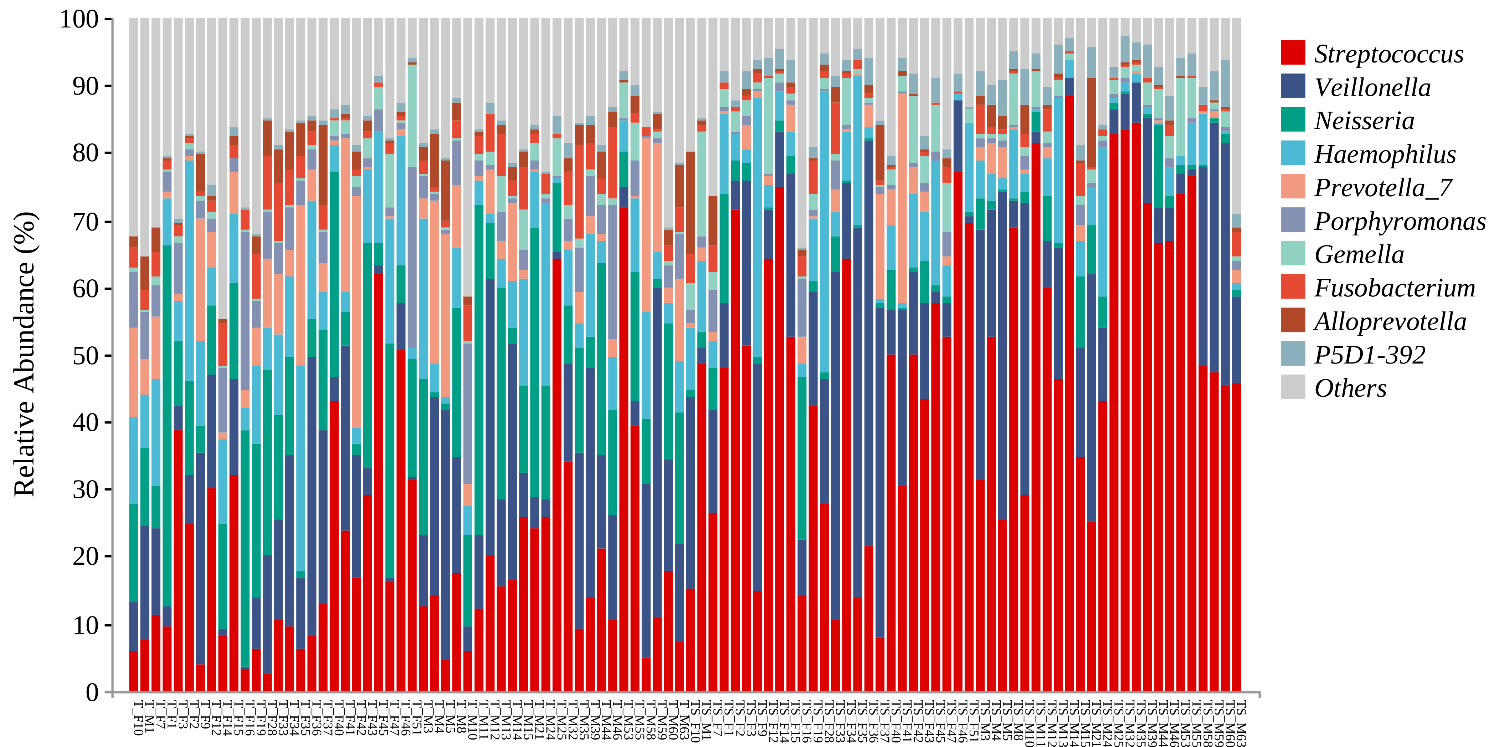


**Supplementary Figure 3** Detailed bacterial community composition at the genus level of each sample. The top 10 dominant bacterial genera in the healthy human oral cavity were *Streptococcus, Veillonella, Neisseria, Haemophilus, Prevotella_7, Porphyromonas, Gemella, Fusobacterium, Alloprevotella,* and *P5D1-392*.


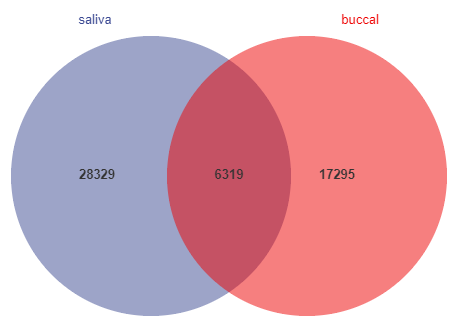


**Supplementary Figure 4** Venn diagram showing the unique and shared ASVs in saliva and buccal mucosal samples.


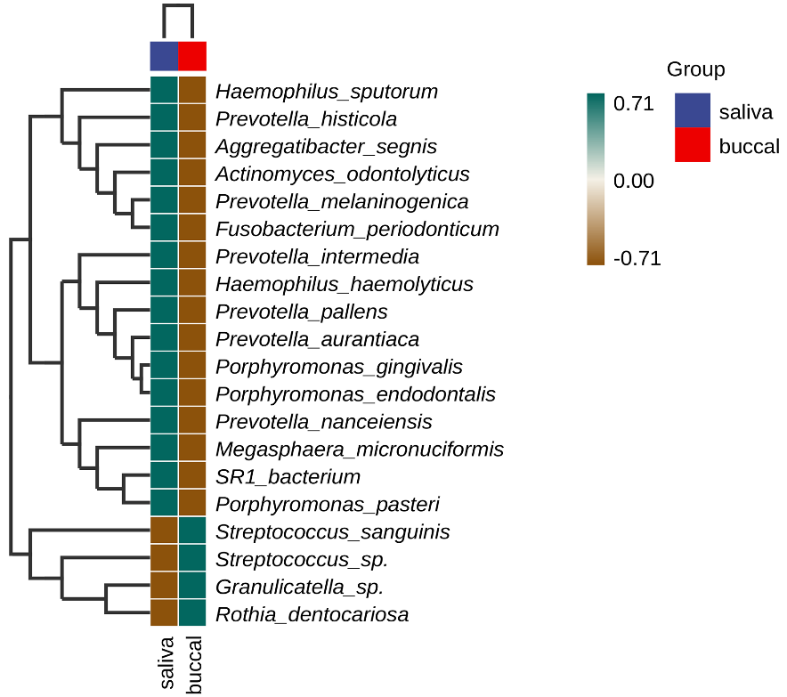


**Supplementary Figure 5** Species variability analysis according to the overall mean values within the group at the species level. A total of 20 bacterial species contained 16 in saliva group and 4 in buccal mucosa group showed a significant difference.


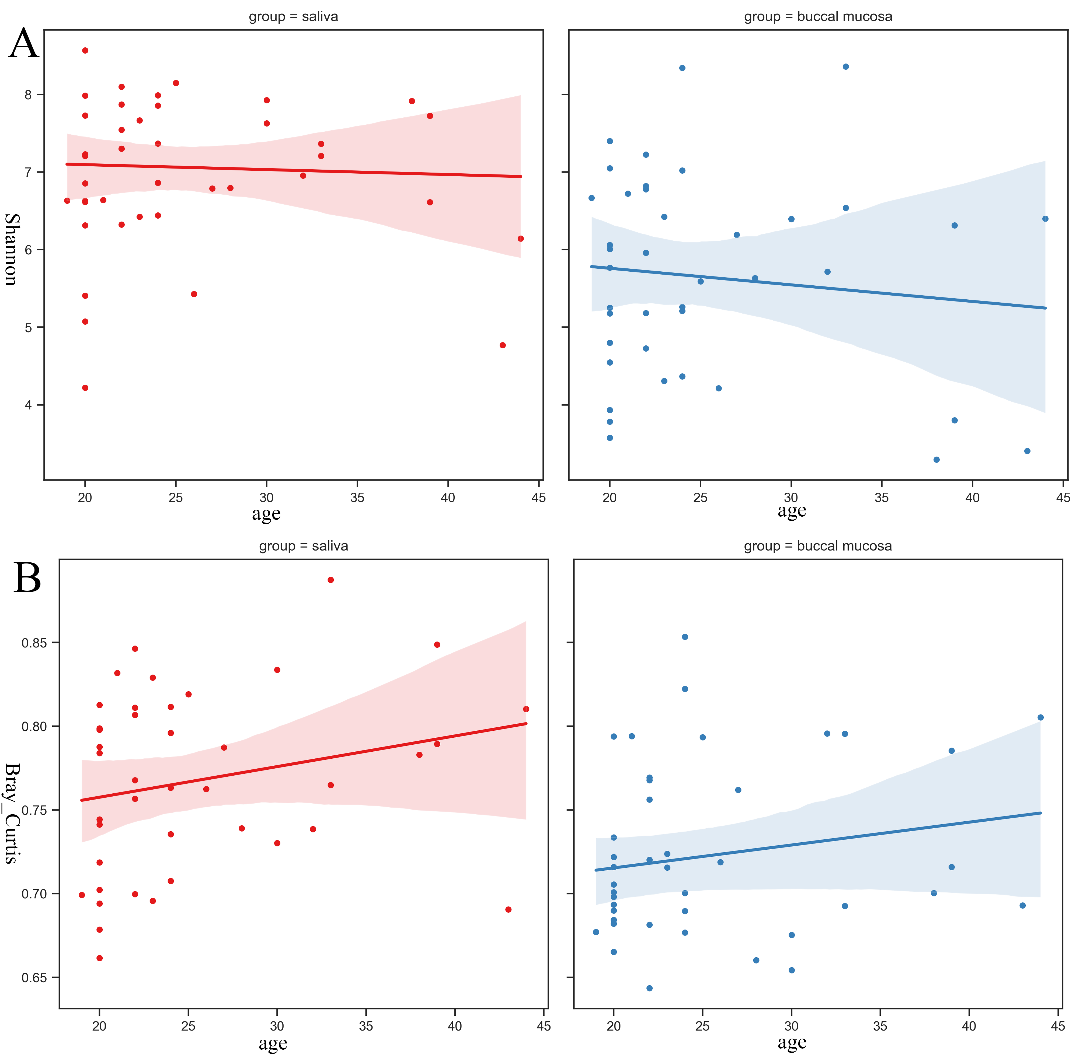


**Supplementary Figure 6** Spearman correlation test was performed for Shannon index, Bray_Curtis distance and the age of subjects. (A) showed no significant correlation between Shannon index and age in salivary and buccal mucosal group (saliva: R=0.087, p=0.59; buccal mucosa: R=-0.0037, p=0.98). (B) showed no significant correlation between Bray_Curtis distance and age in salivary and buccal mucosal group (saliva: R=0.26, p=0.11; buccal mucosa: R=0.19, p=0.23).

**Supplementary Table 1** The α diversity of salivary and buccal mucosal bacterium.

**Supplementary Table 2** The distance matrix of Bray-Curtis dissimilarity index of salivary and buccal mucosal bacterium.

**Supplementary Table 3** The relative proportion of 16 core bacterial genera in saliva and buccal mucosa of all subjects.

**R script of random forest model:**

rm(list=ls()) #clear up, start with a clean session

library(randomForest) #loadding package ‘randomForest’ based on R 3.6.3

otu <- read.csv('all1.csv', row.names = 1) # read ASVs file

otu <- otu[which(rowSums(otu) >= 100), ] # transposition

otu <- data.frame(t(otu))

group <- read.csv('treatment.csv', row.names = 1) #read group file

otu_group <- cbind(otu, group)#combine ASV file and group file

set.seed(123)

select_train <- sample(100, 100*0.7) # divide the data set into training set (70%) and test set (30%)

otu_train <- otu_group[select_train, ]

otu_test <- otu_group[-select_train, ]

set.seed(123)

# perform random forest (generated 500 trees by default)

otu_train.forest <- randomForest(groups ~ ., data = otu_train, importance = TRUE,proximity=TRUE)

plot(margin(otu_train.forest, otu_train$groups), main = 'probability')

train_predict <- predict(otu_train.forest, otu_train)#test train data

compare_train <- table(train_predict, otu_train$groups)

compare_train

sum(diag(compare_train)/sum(compare_train))

test_predict <- predict(otu_train.forest, otu_test)#assess test data

compare_test <- table(otu_test$groups, test_predict, dnn = c('Actual', 'Predicted'))

compare_test

importance_otu <- otu_train.forest$importance

head(importance_otu)#view the importance score of each ASV and identify key ASVs

varImpPlot(otu_train.forest, n.var = min(30, nrow(otu_train.forest$importance)), main = 'Top 30_importance')

importance_otu <- data.frame(importance(otu_train.forest))

head(importance_otu)

write.table(importance_otu, 'importance_otu.txt', sep = '\t', col.names = NA, quote = FALSE)#write table

#order by the value of "Mean Decrease Accuracy"

importance_otu <- importance_otu[order(importance_otu$MeanDecreaseAccuracy, decreasing = TRUE), ]

head(importance_otu)

# choose top30 important ASVs,

otu_select <- rownames(importance_otu)[1:30]

otu_train_30 <- otu_train[ ,c(otu_select, 'groups')]

otu_test_30 <- otu_test[ ,c(otu_select, 'groups')]

set.seed(123)

otu_train.forest_30 <- randomForest(groups ~ ., data = otu_train_30, importance = TRUE)

otu_train.forest_30

plot(margin(otu_train.forest_30, otu_test_top30$groups), main = 'probability')

train_predict <- predict(otu_train.forest_30, otu_train_30)#test train data

compare_train <- table(train_predict, otu_train_30$groups)

compare_train

test_predict <- predict(otu_train.forest_30, otu_test_30) #assess test data

compare_test <- table(otu_test_30$groups, test_predict, dnn = c('Actual', 'Predicted'))

compare_test
